# Oxygen-Releasing Composites: A Promising Approach in the Management of Diabetic Foot Ulcers

**DOI:** 10.3390/polym13234131

**Published:** 2021-11-26

**Authors:** Dong-Jin Lim, Insoo Jang

**Affiliations:** 1 Department of Otolaryngology Head & Neck Surgery, University of Alabama at Birmingham, Birmingham, AL 35294-0012, USA; daniel.djlim@gmail.com; 2 Department of Internal Medicine, College of Korean Medicine, Woosuk University, Jeonju 54987, Korea

**Keywords:** oxygen-releasing material, diabetic complications, diabetic foot ulcer, diabetes

## Abstract

In diabetes, lower extremity amputation (LEA) is an irreversible diabetic-related complication that easily occurs in patients with diabetic foot ulcers (DFUs). Because DFUs are a clinical outcome of different causes including peripheral hypoxia and diabetic foot infection (DFI), conventional wound dressing materials are often insufficient for supporting the normal wound healing potential in the ulcers. Advanced wound dressing development has recently focused on natural or biocompatible scaffolds or incorporating bioactive molecules. This review directs attention to the potential of oxygenation of diabetic wounds and highlights current fabrication techniques for oxygen-releasing composites and their medical applications. Based on different oxygen-releasable compounds such as liquid peroxides and solid peroxides, for example, a variety of oxygen-releasing composites have been fabricated and evaluated for medical applications. This review provides the challenges and limitations of utilizing current oxygen releasable compounds and provides perspectives on advancing oxygen releasing composites for diabetic-related wounds associated with DFUs.

## 1. Introduction

Diabetes is a common and growing disease, affecting nearly 415 million people in the world. Among common diabetes-related complications, lower extremity amputation (LEA) has been recognized as an irreversible complication that usually occurs due to the failure of sufficient preventive care [1]. The risk of LEA in diabetic patients is about 10-fold higher than in the general population [2]. Diabetic foot ulcer (DFU), peripheral neuropathy, and ischemia are usually responsible for LEA. LEA leads to poor quality of patient life, while simultaneously increasing mortality rate. Both immobility and a restricted social life are well-known inconveniences of diabetic patients with LEA [3]. According to a recent study, 1 of 10 patients under major LEA procedures died within 30 days, and male patients were more susceptible regarding mortality [4]. To overcome this unfavorable complication associated with diabetes, it is often required to treat DFUs with elaborative measures. However, the complexity and variation in the origin of the diabetic wound has often resulted in following conventional offloading surgical procedures or offloading treatments with either removable or nonremovable knee-high devices without considering the development of new therapeutic composites [5].

Recent advanced biomaterials for regenerative medicine have also been applied to increase the potential efficacy of wound dressing materials for DFUs. For example, a variety of nanoparticles containing growth factors (e.g., vascular endothelial growth factor; VEGF and basic fibroblast growth factor; bFGF), microRNA (miRNA), and antibacterial agents (e.g., ciprofloxacin) showed improved healing potential in diabetic in vivo models [6,7,8,9]. In addition to these typical bioactive agents, antioxidants have also been incorporated into multiple functional composites for chronic wounds, including diabetic wounds [10,11,12]. Because all diabetic-related complications, including DFUs, have multifactorial etiology, oxygen-releasing composites serve as a source of oxygenation for DFUs and would serve as another essential biomaterial for rescuing chronically impaired wounds. Oxygen supports normal wound metabolism, the synthesis of critical extracellular matrices, and the migration and subsequent proliferation of participant cells such as fibroblasts and macrophages [13]. Moreover, oxygen improves the innate defense mechanism against bacterial invasion by supporting the immune cell-mediated generation of bactericidal oxygen free radicals in physiological conditions [14,15].

In this regard, hyperbaric oxygen therapy (HBOT) has received attention for the past decades and shows positive outcomes. Increased collagen deposition, improved immune activity, and enhanced wound healing have been reported in nondiabetics and DFUs [16]. However, currently available data on the efficacy of HBOT also indicates the variability of adjunctive therapy due to the effect of hyperbaric oxygenation on the damaged tissues. In the hyperbaric oxygen therapy (HBOT) setting, patients receive 100% oxygen directly in a pressured environment and are thus likely to be subjected to central nerve system (CNS) oxygen toxicity [17]. Specifically, overproduction of reactive oxygen species (ROS), formation of peroxynitrite (ONOO^-^) from nitric oxide, and gross retention of carbon oxide (CO_2_) in the brain are thought to cause the oxygen toxicity seizure. At low concentrations, oxygen is also reported to create ROS-mediated wound healing signals, while over-dosage of oxygen supplements can increase oxygen cytotoxicity [18]. Hence, oxygen composites that deliver concentrated oxygen in the atmosphere would become a critical element of an ideal diabetic wound dressing material. This article is intended to discuss current oxygen-releasable compounds and oxygen-releasing composite materials as well as recent fabrication techniques for oxygen-releasing composites capable of topically providing oxygen on diabetic-related wounds associated with DFU.

### 1.1. Diabetic Foot Ulcers (DFUs)

DFUs are one of the most significant diabetes-related complications associated with the hyperglycemic crisis, and the rate of occurrence has remained the same for the past two decades [19]. Diabetic foot ulceration (DFU) characterized by full-thickness penetration of the dermis should be closely monitored to prevent LEA. Several grading systems, including the Wagner system, have been used to characterize DFUs [20]. According to anatomical features, the extent of infection, and the presence of gangrene, the Wagner system can distinguish the degree of diabetic foot ulcerations in diabetic patients and provide either preventive treatment or suitable interventions [21]. The prevalence of DFU was reported as 4% to 10%, while the lifetime risk of a diabetic patient increases up to 25% [22]. Moreover, the recurrence rate of DFU was assumed to be as high as 70% within five years [23]. Peripheral neuropathy, excessive plantar pressure, and repetitive trauma are common causative factors that lead to DFU formation [22]. According to a prevalence study, peripheral neuropathy is common in half of the sixty-year-old type 2 diabetic patients [24]. Likewise, diabetic patients within 25 years of diabetic onsets have symptomatic peripheral neuropathy and subsequent sensory neuropathy that causes the malfunction of protective sensation. Hence, diabetic patients with those diabetic-related complications are prone to experience ultimate physical and thermal trauma [25]. Motor neuropathy is another type of peripheral neuropathy found in diabetic patients, resulting in neuropathic DFU [26]. In combination with sensory loss, the weakened muscles in the foot change the typical foot shape, thereby increasing plantar pressure in the foot, a critical parameter for the development of a neuropathic DFU. The high plantar pressure within diabetic patients is a causative factor, and a 30% reduction in maximum plantar pressure can reduce the risk of developing DFUs [27]. The third causative factor, repetitive trauma, can occur from normal walking activities, through which the foot undergoes an undesired pressure change [28]. The high plantar pressure over time results in neuropathy, deformity, and callus formation [29]. Regarding the risk of LEA during a person’s lifetime with diabetes, peripheral arterial disease (PAD) is another substantial contributing factor. Atherosclerosis, created by the blocking of arteries with fatty deposits, contributes to the development of PAD. The narrowed arteries reduce the blood flow of the heart, brain, and limbs, leading to multiple diabetes complications in diabetic patients [30]. When PAD develops, diabetic patients often feel calf and lower extremity pain, called intermittent claudication. Most diabetic patients with PAD, however, are asymptomatic, meaning that early diagnosis is essential to reduce the chance of developing LEA. Although both DFU and PAD increase the possibility of needing LEA, DFUs are a more critical risk factor than PAD in terms of necessitating LEA. Over 80% of LEA were reported because of nonhealing wounds and ulceration [31].

#### 1.1.1. General Pathobiochemical Hallmarks of Chronic Wounds in the DFU

**Figure 1:**
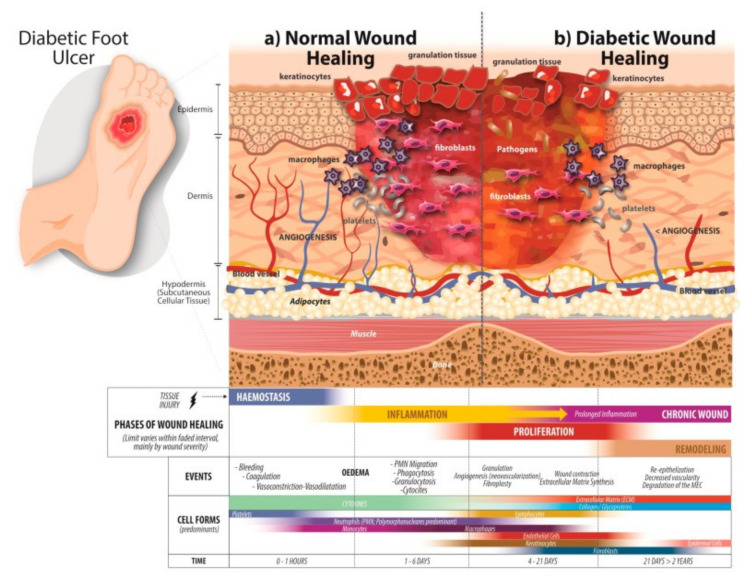
Wound healing phases. (**a**) Normal wound healing phases. In healthy people, wound closure consists of several processes that occur sequentially: rapid hemostasis that involves platelet aggregation to form the platelet plug; an inflammation phase where neutrophils, macrophages, and mast cells release proinflammatory cytokines; wound contraction when inflammation decreases, angiogenesis occurs, keratinocytes and fibroblasts migrate, and the extracellular matrix forms; and, finally, the remodeling phase, where granulation tissue converts into mature scar tissue. (**b**) Diabetic wound healing phases. The wound healing phases are altered, starting with a decrease in fibrinolysis and an imbalance of cytokines. A decrease in angiogenesis due to hyperglycemia and the migration of cells such as keratinocytes and fibroblasts is diminished, causing deficient re-epithelialization; in the same way, poor production of the ECM by fibroblasts contributes to the emergence of DFUs. Reproduced from [32].

In an acute injury, damaged skin can initiate the normal wound healing phases comprised of hemostasis, inflammation, proliferation, and remodeling phase, whereas lower extremity wounds are characterized by impaired wound healing phases (Figure 1) [32]. Delayed wound healing is associated with several pathophysiological changes in diabetic wounds. Elevated inflammatory cytokines (e.g., IL-1β and TNF-α), lowered circulating endothelial progenitor cells (EPCs), and reduced stromal cell-derived factor 1 (SDF1) were consistently found in diabetic wounds [33,34]. Lack of sufficient concentrations of the growth factor, granulocyte macrophage-colony stimulating factor (GM-CSF), was also observed in diabetic wounds [35]. In addition, Matrix metalloproteinases (MMPs) and tissue inhibitors of metalloproteinases (TIMPs), which play a significant role in the remodeling of ECM, are not balanced in chronic diabetic wounds [36,37]. Moreover, in the state of diabetic neuropathy, glucose levels in the wound area are likely to rise without sufficient blood supply. High glucose in the wound area delays the re-epithelization stage of wound healing by impairing keratinocyte migration [38]. According to several recent studies, p38/mitogen-activated protein kinase (MAPK), which regulates keratinocyte migration, was altered in high glucose conditions [39,40].

#### 1.1.2. General Risk Factors Associated with DFU for Preventing LEA

**Figure 2:**
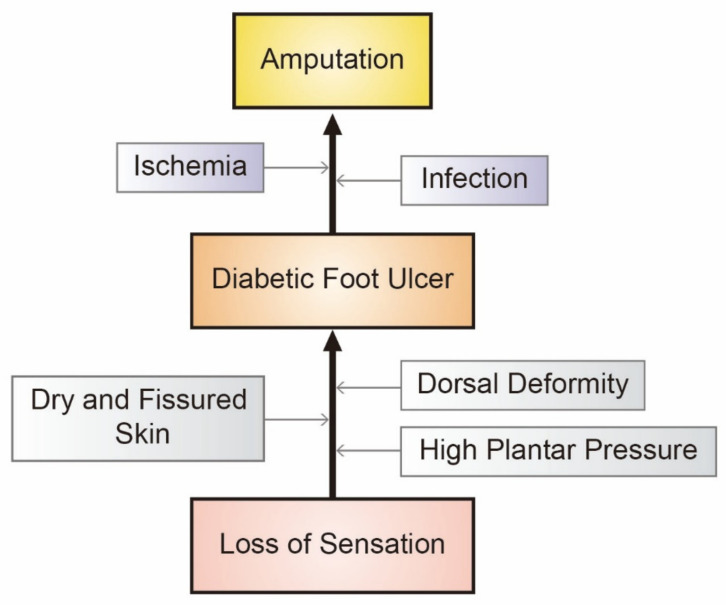
Major causal factors that lead to lower extremity amputation in diabetics.

To prevent ultimate amputation, the major causes of DFUs should be managed. Both ischemia and infection are considered critical causal factors that unfortunately lead to LEA (Figure 2) [41]. In diabetic ischemic ulcers, hypoxia is responsible for reduction of hypoxia-inducible factor-1 (HIF-1), which is a transcription factor that mediates oxygen homeostasis [42]. This reduction of HIF-1 activation thereafter contributes to the delayed wound healing seen in DFUs [42]. Moreover, hyperglycemia destabilizes HIF-1α, which is the regulatory subunit of HIF-1 [43]. In a recent in vitro study, human dermal fibroblasts (HDFs) and human dermal microvascular endothelial cells (HDMECs) secreted reduced HIF-1α in high glucose media compared to normal cells grown in regular glucose media [44]. The increased pressure of the plantar aspect of the foot observed in diabetic patient groups is another casual factor [45]. High plantar foot pressure leads to ulceration in diabetic populations, regardless of ethnicity, and diabetic neuropathy also independently affects the extent of ulceration [46].

In addition, diabetic foot infection (DFI) is a well-known risk factor for LEA [31]. Multiple causative factors such as diabetic immunopathy, diabetic neuropathy, and diabetic angiopathy, and impaired skin antimicrobial defense mechanisms in both physical and biochemical manner induce the polymicrobial biofilm infections [47]. Following chronic infections associated with reduced blood flow in peripheral artery disease, the normal recruitment of immune cells fails, and high glucose levels in the peripheral blood vessels retard the proper function of neutrophiles, which is a critical step of the proper host antimicrobial defense [48]. Gram-positive bacteria such as *Staphylococcus aureus* and *Streptococcus* spp. were commonly found in mid-infected diabetic wounds, while both Gram-positive and Gram-negative bacteria were found in the case of severe infections [49]. Moreover, multidrug-resistant (MDR) microorganisms are easily found in polymicrobial infected DFUs, thereby reducing the efficacy of available antibiotic treatments [50]. Such hard-to-kill pathogens are the primary reason for poor clinical outcomes when treated with conventional antibiotic treatment for DFIs [51].

#### 1.1.3. Topical Ulcer Treatment of Preventing the DFU

While avoiding the risks mentioned above, appropriate topical ulcer treatments are essential to reduce the risk of LEA. To date, commonly utilized topical ulcer treatments include debridement, wound dressing, and antimicrobial wound dressing. All wound dressings can absorb excessive exudates from wounds, creating the favorable microenvironment that initiates the wound healing process. Based on the physical types of wound dressings, four significant forms are currently found in the market: hydrogels, hydrocolloids, foams, and films [52]. However, for treating chronic wounds, including DFUs, more functional wound dressings are more attractive. A variety of wound care products are already commercially available using advanced matrices and bioactive molecules (Table 1). For example, natural polymers such as hyaluronic acid and alginate are frequently used materials. Collagen is also a well-utilized advanced matrix material intended for promoting the wound healing process. Likewise, human cellular wound dressings are used for supporting epidermal reconstitution. The extracellular matrices (ECMs) facilitate the normal wound healing processes. The ECM interacts with cells capable of remodeling the damaged tissues. In addition, recent studies indicate that the fragmented ECM molecules also act as singling cues for governing the healing processes [53,54]. Several bioactive molecules have been widely used in the development of wound dressings for DFUs. For example, inorganic antimicrobial agents such as silver and iodine are utilized without the risk of antibiotic resistance, in contrast to conventional antibiotics which can sometimes be limited by antibiotic resistance.

## 2. Oxygen Releasable Compounds: Different Approaches for the DFU

Oxygen releasable compounds can achieve a topical oxygen delivery to chronic wounds. In general, oxygen-releasing composites have been fabricated by embedding oxygen releasable compounds such as solid peroxides, liquid peroxides, and fluorinated molecules into biocompatible matrices. Through different triggering mechanisms, these oxygen-carrying compounds can release oxygen (Table 2). This section explains the different types of oxygen releasable compounds commonly used to give an insight on the development of topical oxygen-based methods for diabetic wounds repair.

## 3. Oxygen in Diabetic Wound Repairs

**Figure 3:**
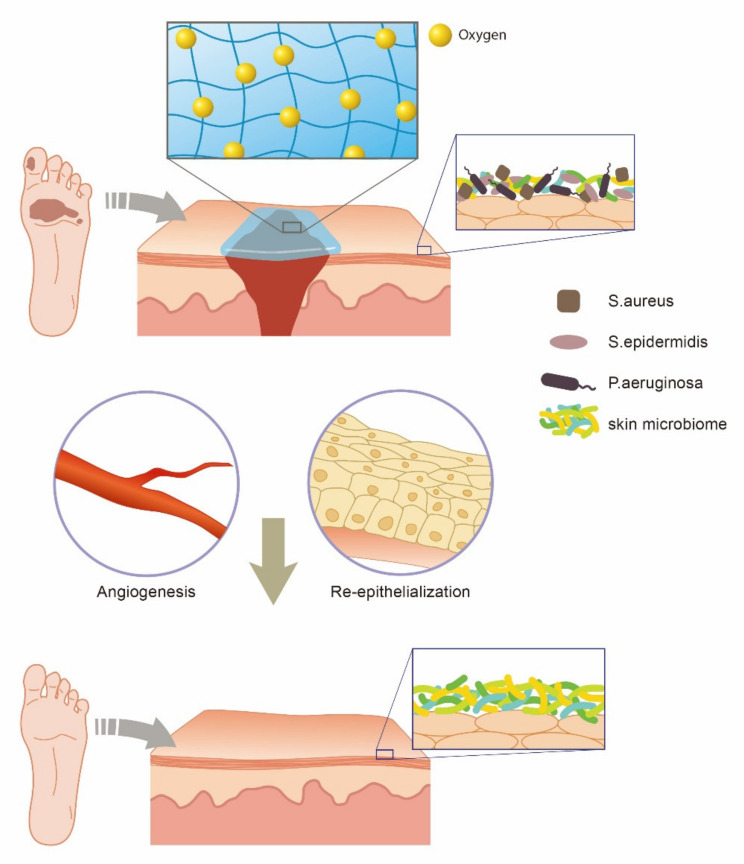
Role of oxygen in the management of DFU.

Oxygen is another critical factor for increasing the successful healing of chronic wounds. The oxygenated microenvironment during angiogenesis improves the wound healing process (Figure 3). Oxygen also promotes procollagen maturation and collagen deposition for proper epithelialization [60,61]. Even at the molecular level, hypoxia in wounds can initially stimulate collagen fiber formation through inactivated HIF-1 mediated procollagen hydroxylases, thus supporting the fact that proper oxygen tension is essential for the completion of the wound-healing process [62]. While promoting the healing process, increased oxygen tension also helps enhance the antibacterial activity of leukocytes recruited near chronic wounds [63].

In this regard, hyperbaric oxygen therapy (HBOT) provides pure oxygen to diabetic patients in a pressurized oxygen chamber and has been clinically used to manage diabetic foot ulcers for the last decades [64,65]. In a randomized, double-blinded, and placebo-controlled clinical trial performed at a Sweden hospital with ninety-four patients with above Wagner grade 2, the HBOT patient group treated with at least 35 HBOT sessions during an eight-week study showed improved healing of ulcers [65]. In another randomized clinical trial, the significant amputation rate in diabetic patients with severe foot ulcer was reduced when treated with systemic HBOT. Only 8.6% of subjects with systemic HBOT led to major amputation compared to 33% in the nontreated group (*p* = 0.016) [66]. HBOT also demonstrated improved healing rates and quality of life in diabetic foot ulcers when applied for several months [67].

However, HBOT has several drawbacks in terms of clinical complications associated with repetitive use and accessibility. Common difficulties with pressured oxygen chambers include middle ear pain, cranial sinus pain, and teeth pain. Almost 10% of subjected patients had reported such pain, and over half of the patients terminated the HBOT [68]. In addition to the clinical complications, some patients experienced claustrophobia due to the enclosed chamber. Because HBOT affects the whole body during treatment, pressure-associated side effects including middle ear barotrauma (MEB), sinus/paranasal barotrauma, dental barotrauma, and pulmonary barotrauma are common [17]. Moreover, artificial oxygen tension created in the enclosed chamber can induce central nervous system (CNS) oxygen toxicity [69].

## 4. Examples of Oxygen-Releasing Composites in the Tissue Engineering

Based on the oxygen releasable compounds mentioned above, different types of oxygen-releasing composites have been developed (Table 3). Recent oxygen-releasing composites have mainly been studied to either create a normoxic cellular microenvironment in pathological tissues or sustain oxygen delivery for supporting tissue-engineered constructs.

### 4.1. Hydrogen Peroxides: Orangic Oxygen Releasing Compounds

A study used a double encapsulated oxygen delivery system to achieve sustained oxygen delivery while reducing the cytotoxicity of hydrogen peroxide [70]. The first encapsulated layer was made from H_2_O_2_ and biocompatible poly(D,L-lactide-co-glycolide) (PLGA) polymer. Then, a covalently cross-linked catalase-alginate polymer was used to enclose the shelled H_2_O_2_ composite, leading to prolonged oxygen delivery without cellular cytotoxicity. Silica gel, polyvinylpyrrolidone (PVP), and small molecules (e.g., urea, sodium carbonate, and sodium borate) entrapped H_2_O_2_ molecules within the composite [79,80]. In such silica-based encapsulation, divalent metal ions, Mg^2+^ and Ca^2+^, also contributed to the release of H_2_O_2_ from silica hydrogels [81]. Such engineerable characteristics of materials that modulate the extended H_2_O_2_ delivery indicate that liquid H_2_O_2_ molecules hold great potential for the realization of topical oxygen delivery in diabetic wounds repair.

### 4.2. Solid Peroxides: Inorangic Oxygen Releasing Compounds

Compared to liquid hydrogen peroxide, solid peroxides are flexible to achieve controlled oxygen delivery through encapsulating them within biocompatible materials such as PLGA and polydimethylsiloxane (PDMS). Harrison et al. created SPC-encapsulated PLGA films that release oxygen for 70 h, contributing to the prevention of graft necrosis as shown in mice skin flaps [82]. An implantable silicone encapsulated CPO, exhibiting much more extended oxygen release for transplanted islets, was developed [83]. In this study, authors fabricated a CPO (25% wt/wt)-embedded PDMS disc to rescue beta-cells, which produced insulin in response to glucose. Because beta-cells reside in the core of islets, they need to receive significant oxygen continuously while responding to physiological glucose levels. A CPO-embedded PDMS disc demonstrated long-term oxygen delivery for four weeks with the highest oxygen delivery in the first week, supporting enhanced viability and functionality of the MIN6 β cell line and rat pancreatic islets in vitro for 14 days [84]. In other studies, antioxidant-doped polyurethane (PU) scaffold (PUAO) encapsulated CPOs were utilized [85,86]. The PUAO-CPO scaffolds released oxygen and prevented skin flip necrosis in mice in vivo model up to nine days.

### 4.3. Perfluorocarbons: Passive Oxygen Releasing Compounds

Perfluorocarbons are halogen-substituted nonpolar carbon-based oils. Perfluorocarbon (PFC) emulsions are typical forms of enhanced oxygen delivery [87]. For example, Fluosol-DA-20% obtained FDA approval to prevent coronary ischemia and demonstrated enhanced oxygen delivery because of large surface ratios of the PFC emulsions, facilitating oxygen charges subjected to Henry’s Law (in PFCs).

## 5. Topical Oxygen-Releasing Composites in the Management of the DFU

Based on our previous reviews on oxygen biomaterials, this section describes how such oxygen-releasing composites help heal wounds (e.g., chronic wounds) commonly observed in early LEA. Although most scaffolds are not specific for DFUs, these studies give a perspective on the ongoing development of functional oxygen-releasing composites for diabetic-related complications.

### 5.1. Topical Delivery of Oxygen: Significance of Repeated Oxygen Supplements

As an alternative to using sophisticated methods involved in HBOT, topical hyperbaric oxygen (THO) treatment using a disposable or reusable limb chamber attached to an oxygen tank has been studied. However, there is controversy regarding the efficacy of THO on diabetic wounds exhibiting delayed healing [88]. Another approach toward improved wound healing is transdermal oxygen therapy (TOT). A portable miniature device (EPIFLO^®^) capable of producing oxygen from the ambient air delivers nearly pure oxygen around diabetic wounds for 15 days [89]. The concept of TOT was also studied using excisional pig dermal wounds where repeated TOT treatment via topical oxygen device accelerated wound closure in the early post-wound phase [90]. According to a nine-month follow-up study, seven surgeons performed TOT treatment on the wounds (*n* = 58) of 32 patients. About 73% of the wounds from fifteen participating patients were completely cured. Even though ten wounds resulted in no positive outcome during the TOT treatment, TOT has a beneficial role in facilitating wound healing without complications overall [91].

### 5.2. Potential Examples of Viable Oxygen-Releasing Composites for the DFU

An oxygen-delivering hydrogel, called methacrylamide chitosan, modified with perfluorocarbon chains (MACF) hydrogel, was fabricated and evaluated for use as a topical oxygen deliverable wound dressing [91]. The MACF hydrogel showed 100% oxygen saturation within 10 min. The oxygenated MACF showed the best re-epithelialization compared to controls (no hydrogel, non-oxygenated MACF, unmodified methaacrylamide (MAC), and non-oxygenated MAC). Further metabolomic analyses also indicated that MACF hydrogel promoted the metabolic expression associated with wound healing, followed by collagen synthesis in the granulation bed. In this study, the MACF hydrogel was replaced every two days and observed for eight days.

Another oxygen-generating hydrogel (OG hydrogel) was developed by incorporating glucose oxidase (GOx, EC 1.1.3.4) and peroxidase (PO, EC 1.11.1.7) within either PVA or PVP-based hydrogel via γ-irradiation cross-linking [92]. The prepared OG hydrogel produced oxygen in response to glucose and released iodine from potassium iodine. This composite contributed to rapid diabetic wound healing while combatting microbial wound infections. A paper-based oxygen-releasing platform was also studied [88]. Using the ability of manganese dioxide (MnO_2_) to convert H_2_O_2_ into oxygen and water, the authors fabricated a PDMS microfluidic device with parchment paper onto which MnO_2_ were selectively deposited in the designated catalytic regions of the microfluidic device. In this device, generated oxygen from the catalytic regions was freely permeated through the installed parchment paper, supporting cell survival without harmful opposite-side flowing H_2_O_2_ cytotoxicity. Because new wound dressings can replace old ones daily, such repetitive replacements would boost the benefits of oxygen therapy while maintaining the advantage of oxygen biomaterials in practical wound management.

### 5.3. Antibacterial Activities of Oxygen-Releasing Composites

In the process of wound healing, oxygen can contribute not only to the production of energy essential for tissue regeneration, but also to the establishment of anti-microbial defense mechanisms [93]. Not only can solid peroxides provide oxygen in contact with water, but they also exhibit antibacterial activity due H_2_O_2_ production. In a study using polycaprolactone (PCL) nanofibers as CPO carriers, the CPO-PCL nanofibers inhibited the growth of *Escherichia coli* (*E. coli*) and *Staphylococcus epidermidis* (*S. epidermidis*) at the initial incubation period, during which embedded CPOs were rapidly released [94]. Similarly, an oxygen-releasing hybrid polymeric nanofiber scaffold composed of poly(glycerol sebacate) (PGS) and poly(*ε*-caprolactone) (PCL) was studied [95]. In this study, nanofibers with more fine CPO nanoparticles exhibited a sustainable oxygen release for up to one week. In addition to antibacterial activity against *Staphylococcus aureus* (*S. aureus*), the CPO-embedded PGS/PCL nanofibrous also showed gradual viability recovery of primary bone-marrow-derived mesenchymal stem cells (BM-MSCs).

### 5.4. Perspecitve and Future Directions for Oxygen-Releasing Composites

**Figure 4:**
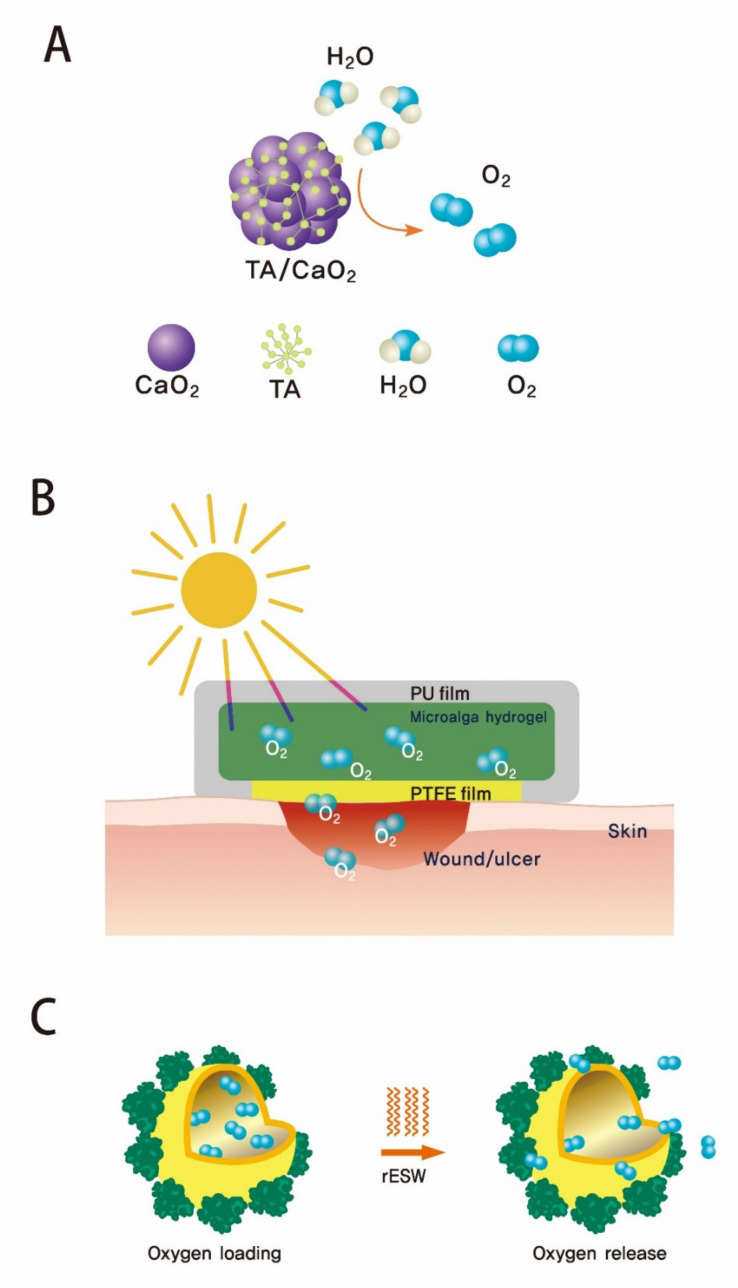
Recent development of oxygen-releasing composites applicable for DFU treatments. (**A**) Tannylated calcium peroxide nanoparticle. In this study, tannic acid was used to coordinate the bridge between calcium ions [96], (**B**) Novel microalga-gel patch (AGP). The fabricated patch was filled with gel beads containing active *Synechococcus elongatus* (*S. elongatus*) PCC7942, a unicellular cyanobacterium that produces oxygen for diabetic chronic wounds [98], and (**C**) Oxygen-loaded nanperfluorocarbon (Nano-PFC). The radial extracorporeal shock wave (rESW) was employed to trigger the release of oxygen from a human serum albumin (HSA)-stabilized perfluorocarbon (perfluoro-15-crown-5-ether) emulsion [97].

In the perspective of prolonged topical oxygen delivery for DFUs, it is worth paying attention to recent studies showing both developments of new functional materials and the utilization of new oxygen sources (Figure 4). A study directly fabricated an oxygen-releasing composite by stabilizing the formed CPO nanoparticle with tannic acid (TA) [96]. The authors created spherical CPO aggregates intermingled with tannic acids with sizes of 25–31 nm. Compared to the normal several micrometer sizes of CPO particles, such a stable small size of oxygen-releasing composites increased the application of solid peroxides in the development of functional composites for a variety of tissue-engineering applications including topical oxygen delivery for DFUs. Another strategy that effectively delivers oxygen applies to the concept of on-demand drug delivery, which has been one of the more interesting topics in the field of pharmaceutical sciences. Utilizing the principle of radial extracorporeal shockwave therapy (rESWT), an in vivo study demonstrated that the radial extracorporeal shock wave (rESW) can initiate the release of oxygen from oxygen-loaded nano-perfluorocarbon (Nano-PFC) emulsions [97]. The authors showed rESW-responsive oxygen release from the oxygen-saturated emulsions. Instead of using conventional oxygen-releasable compounds, a recent innovative study employed a living microorganism to serve as an oxygen provider under light [98]. While there are a lot of practical challenges required for becoming a clinically applicable technology, such innovated strategies give us new insight for developing more ideal oxygen-releasing composites that have the potential to manage diabetic chronic wounds.

## 6. Conclusions

Ineffective management of DFUs can lead to terminal LEA. As previously described, all types of diabetic foot ulcers result from numerous risk factors such as peripheral neuropathy, peripheral vascular disease, foot deformities, arterial insufficiency, trauma, and impaired resistance to infection. However, there is no ideal wound dressing suitable for all diabetic patients suffering from pathologies with different sets of causes. A study indicated that almost 12% of all diabetic patients with DFUs will require amputation, and most patients who have undergone LEA are still at risk of developing ulcers in the contralateral limb [99]. In this regard, there is an ongoing need for developing more functional wound dressings tailored to diabetic patients. Topical ulcer treatment with oxygen would be a potential therapy for treating DFUs in the healing stages, which has already been impaired by diabetes. Because of the unique drawbacks of each oxygen compound, few studies have been reported to develop such functional wound scaffolds with oxygen-releasing composites. Oxygen-carrying perfluorocarbons in lipophilic carriers have limited shelf life, and safety issues associated with both used perfluorocarbons themselves and carriers [56,100]. Meanwhile, solid peroxides cannot avoid undesired byproducts (e.g., hydroxide, H_2_O_2_ in high level) during the oxygen generation. Such limitations in oxygen releasing compounds, however, may be resolved by incorporating advanced fabrication techniques and new functional biomaterials that exhibit sustained oxygen delivery from these compounds while minimizing or neutralizing reactants. Further advancement in fabrication techniques as well as in the development of functional materials for oxygen-releasing composites will allow us to present a viable option for better diabetic chronic wound management that recuses vulnerable diabetic patients to both DFUs and LEA.

## Figures and Tables

**Table 1:** The typical examples of commercially available topical wound dressings for DFU ^1^.

Commercial Dressing	Company	Composition	Main Characteristics
Bionect^®^	BioScience	0.2% of sodium salt of hyaluronic acid	Easy to use Reduces the incidence of high-grade skin reactions Reduces wound severity
Unite^®^ Biomatrix	Synovis Orthopedic and WoundCare, Inc.	Non-reconstituted collagen	Collagen dressing helps maintain wound bed in healing phase Allows for healthy granulation tissue and wound closure Absorbs excess exudate, thus reducing dressing changes Easily conforms to the wound bed Strong and durable
BGC Matrix^®^	Mölnlycke Health Care US, LLC	Collagen and advanced carbohydrate beta-glucan	Protects underlying tissue from external contamination Provides structural support for new cell growth Adherent, flexible and conformable Minimizes protein and water loss Collagen aids in hemostasis Minimizes pain
Promogran Prisma^®^ Matrix	Systagenix	Collagen, oxidized regenerated cellulose (ORC), and silver-ORC matrix	In the presence of exudate, the matrix transforms into a biodegradable gel Provides protection from infection and an optimal healing environment Designed to “kick start” the healing process in stalled wounds Biodegradable gel is soft and conformable Can be used under compression therapy Nontoxic and non-irritating Easy to use
Dermacol/Ag™ Collagen Matrix Dressing with Silver	DermaRite Industries	Collagen, sodium alginate, carboxyl methyl-cellulose, ethylenediamine-tetraacetic acid (EDTA) and silver chloride	Transforms into a soft gel sheet when in contact with wound exudates Maintains a moist wound environment and creates ideal conditions for healing Antimicrobial silver chloride prevents colonization of the dressing Easy to use
Fibracol^®^ Plus Collagen Wound Dressing with Alginate	Systagenix	Collagen and calcium alginate fibers wound	Structural support of collagen with gel forming properties of alginates Maintains a moist wound environment and creates ideal conditions for healing Adherent, flexible and conformable Sterile and soft
Aquacel Hydrofiber^®^ Wound Dressing	ConvaTec	Antimicrobial hydrofiber containing carboxymethyl cellulose with ionic silver	Absorbs wound fluid and creates a soft gel, which maintains a moist wound environment Absorbs and retains exudate and harmful components such as bacteria contained within exudate, directly into its fibers Helps reduce pain and trauma upon dressing removal Conforms to the wound surface Used on moderately and highly exuding chronic wounds
Regranex^®^ Gel	Healthpoint Biotherapeutics	Human recombinant PDGF-BB ^2^ incorporated in aqueous sodium carboxymethylcellulose	Stimulates wound healing processes and aids in creation of granulation tissue Only FDA-approved topical agent with platelet-derived growth factor Promotes the recruitment and proliferation of chemotactic cells Stimulates wound closure Easy to use
MediHoney^®^ Adhesive Honeycolloid Dressing	Derma Sciences, Inc.	80% active Leptospermum honey with colloidal alginate	Maintains effectiveness even in the presence of wound fluid, blood, and tissue For wounds with light to moderate amounts of exudates Pad forms a gel as it warms up and contacts wound fluid Promotes a moisture-balanced environment conducive to wound healing Helps wounds that have stalled healing High osmolarity cleanses Helps lower overall wound pH Non-toxic, natural, safe and low-cost
MediHoney^®^ Calcium Alginate Dressing	Derma Sciences, Inc.	Contains 95% active Leptospermum honey with calcium alginate	As wound fluid enters the dressing, honey is released while the dressing forms a gel Maintains effectiveness even in the presence of wound fluid, blood and tissue Promotes a moisture-balanced environment conducive to wound healing Highly osmotic and helps to reduce overall wound pH For wounds with moderate to heavy amounts of exudates Non-toxic, natural, safe, and easy to use
Algisite^◇^ M Calcium Alginate Dressing	Smith & Nephew, Inc.	Calcium-alginate	Forms a gel that absorbs exudate when in contact with wound Helps prevent scar formation and promotes wound contraction Allows gas exchange necessary for a healthy wound bed Low-adherence reduces trauma at dressing changes Conforms to wound contours Low fiber shed Easy to remove
Sorbalgon^®^	Hartman USA, Inc.	Calcium alginate	Forms a hydrophilic gel on contact with wound exudate Maintains integrity while dry or wet Highly absorbent Easy to remove Latex-free
Kaltostat^®^ Dressing	ConvaTec	Sodium and calcium salts of alginic acid	In the presence of exudate or other body fluids containing sodium ions, the fibers absorb liquid and swell Calcium ions promote the dressing to take on a gel-like appearance Facilitates wound healing providing a favorable micro-environment Easy to use
Tegaderm™ High Gelling Alginate Dressing	3 M Health Care	Polyurethane dressing containing alginate	Forms a gel-like consistency as it absorbs exudate to provide a moist healing environment Completely gels with saturation for easy removal from fragile tissue by gentle irrigation Easily irrigated from the wound bed when saturated Highly absorbent and conformable
GranuDerm™ Sentry™	Acute Care Solutions, LLC	Alginate hydrocolloid with polyurethane	Breathable film membrane surrounds the wound site Promotes wound repair Visually signals dressing changes Water, dirt, and germ proof Reduces dressing changes Extended wear time Prohibits leakage
Biatain^®^ Heel Foam Dressing	Coloplast Corp.	3-D non-adhesive foam of polyurethane	Foam absorbs and retains wound exudate to control moisture balance in wound Absorbs low-to-high wound exudate levels and protects the heel Decreases wound area and prevents skin maceration Soft and beveled edges make dressing more comfortable for patient Longer wear time for fewer dressing changes Low risk of leakage or maceration Safe and effective
Biatain Ibu Foam Dressing Non-adhesive	Coloplast Corp.	Combination of polyurethane-foam, polyurethane film, polyethylene and ibuprofen	Combines moist wound healing with an active pain reliever Releases ibuprofen evenly into the wound Helps to ease pain from the wound during wear and when changing the dressing Promotes wound repair Easy to use
MANUKAhd^®^	ManukaMed USA, Inc.	Polyurethane foam and film in backing and an absorbent dressing pad of polyacrylate polymers impregnated with ManukaMed^®^ honey	100% active medical grade Manuka^®^ honey Gentle on wounds promoting wound healing Forms gels in contact with exudate Fluid permeable and dry touch
DuoDERM^®^ CGF^®^	ConvaTec	Polyurethane foam	Promotes granulation and facilitates autolytic debridement Can be easily and gently molded into place Use on lightly to moderately exuding acute and chronic wounds Minimize skin trauma and disruption of healing Can be worn for up to seven days Allows observation of the healing process due to its transparency
SOLOSITE^®^ Conformable Wound Gel Dressing	Smith & Nephew, Inc.	Polyurethane and polyethylene hydrogel	Creates a moist wound healing environment Keeps gel in intimate contact with wound surface Absorbs excess exudate thus reducing dressing changes Noncytotoxic and nonsensitizing
Silverlon^®^ Island Wound Dressing	Argentum Medical, LLC	Polyurethane film containing silver	Non-adherent wound contact layer Provides effective protection against microbial contamination Permits passage of wound exudate Stimulates wound repair Easy to apply
Allevyn	Smith & Nephew, Inc.	Polyurethane films combined with polyurethane foam containing 5% silver sulphadiazine.	Minimizes pain to the patient and trauma to the wound during dressing changes Rapid and sustained (seven days) antibacterial action Absorbs, retains, and transpires exudate to provide enhanced fluid management Provides a moist wound environment for the promotion of faster closure Stays in place for up to seven days
Meliplex Ag	Molnlycke Heath Care	Polyurethane foam containing a silver compound (silver sulphate)	Vapor-permeable Waterproof film to absorb exudate Maintains a moist wound environment
Ligasano	Ligasano	Honeycomb-polyurethane foam	Economic and manageable Absorbs high amounts of exudate without dehydrating the wound bed Creates a moist and warm wound environment Antiseptic and cleans the wound without sticking to the wound Stimulates local blood circulation in the wound

^1^ This table is reformatted from [52] with permission, copyright Elsevier, 2013. ^2^ PDGF-BB; Platelet-derived growth factor (PDGF) two B subunits.

**Table 2:** Examples of oxygen releasable compounds and their characteristics.

Compound	Descriptions	Ref.
Calcium peroxide	Released by hydrolytic decomposition Reported saturated concentration: 22 mg/L	[55]
Magnesium peroxide	Released by hydrolytic decomposition Reported saturated concentration: 44 mg/L	[55]
Sodium percarbonate	Released by hydrolytic decomposition Reported saturated concentration: 31 mg/L	[55]
Perfluorodecalin (PFD) (C_10_F_18_)	Oxygen solubility: 403 mL/L_PFD_	[56]
Perfluorooctylbromide (PFOB) (C_8_BrF_17_)	Oxygen solubility: 527 mL/L_PFD_	[56]
Hydrogen peroxide	Converted by blood, catalase, and Horseradish peroxidase	[57,58,59]

**Table 3:** The oxygen-releasing composites from different oxygen releasable compounds.

Compound	Oxygen-Releasing Composite	Descriptions	Ref.
Hydrogen Peroxide	H_2_O_2_-loading poly(D,L-lactide-co-glycolide) (PLGA) particle	Catalase was immobilized alginate used for detoxifying H_2_O_2_.	[70,71]
	H_2_O_2_-incoporating polyvinylpyrrolidone (PVP)/poly(D,L-lactide-co-glycolide) (PLGA) core-shell microparticle	Catalase was covalently incorporated onto the surface of microparticles.	[72]
Calcium Peroxide (CPO)	CPO-loading poly (L-lactic acid) (PLLA) nanoparticle	Catalase was grafted onto the surface of hollow nanoparticles. Nano CPOs were loaded.	[73]
	CPO-encapsulated alginate microcapsule	Because of calcium, alginates were immediately cross-linked.	[74]
	CPO-mediated thiolated gelatin	Thiolated gelatins formed a cross-linkable hydrogel due to CaO_2_-mediated oxidative cross-linking reaction.	[75]
Sodium percarbonate (SPO)	CPO-loading poly (L-lactic acid) (PLLA) nanoparticle	Catalase was grafted onto the surface of hollow nanoparticles.	[73]
	CPO/SPO-PVA and PCL film	In the final contrast, a gelatin layer was served as decomposing H_2_O_2_ through manganese chloride (MnCl_2_).	[76]
Perfluorocarbon	Nano-sized perfluorocarbon ^1^ stabilized by human serum albumin (HSA)	HSA-stabilized nano-emulsion PFC materials have small size in diameter (~80 nm).	[77]
	Perfluorodecalin (C_10_F_18_) and perfluoro-*n*-tripropylamine, (C_3_F_7_)_3_N, based-emulsion	(C_3_F_7_)_3_N was used for stabilizing the final emulsion	[78]

^1^ Perfluoro-15-crown-5-ether.
